# Supplementary material for: The role of direct air capture in achieving climate-neutral aviation
Source: Nat Commun. 2025 Jan 11;16:588. doi: 10.1038/s41467-024-55482-6 (PMC11724844; doi:10.1038/s41467-024-55482-6)
Supplement: Supplementary file 1 — Supplementary Information [file 41467_2024_55482_MOESM1_ESM.pdf]

Supplementary Information

The role of direct air capture in achieving climate-neutral aviation

Nicoletta Brazzola[1*], Amir Meskaldji[1], Anthony Patt[1], Tim Tröndle[1], Christian Moretti[1,2]

*Email: Nicoletta.brazzola@usys.ethz.ch

1. Institute for Environmental Decisions, ETH Zürich, 8092 Zürich, Switzerland

2. PSI Center for Energy and Environmental Sciences, Laboratory for Energy Systems Analysis, 5232 Villigen, Switzerland

Supplementary items list

Supplementary Tables

Supplementary Table 1 – Input parameters for cost calculations

Supplementary Table 2 – Mass flows

Supplementary Table 3 – Input parameters for energy consumption

Supplementary Table 4 – Input parameters for ticket price calculations

Supplementary Table 5 – Input parameters for additional scenarios

Supplementary Figures

Supplementary Figure 1 – Absolute emissions

Supplementary Figure 2 – Direct Air Capture rates

Supplementary Figure 3 – Energy consumption

Supplementary Figure 4 – Total cost

Supplementary Figure 5 – Change in ticket price in 2030

Supplementary Figure 6 – Cost under different technology configurations

Supplementary Figure 7 – Total Cost under different demand scenarios

Supplementary Figure 8 – Breakdown costs under different demand scenarios

Supplementary Figure 9 – Cumulative difference in cost with business-as-usual

Supplementary Figure 10 – Emissions and cost in 2060

Supplementary Figure 11 – Impact of electricity and fossil kerosene cost

Supplementary Figure 12 – Impact of different policy assumptions

Supplementary Figure 13 – Sensitivity analysis for climate neutrality target

Supplementary Tables

Supplementary Table 1 Input parameters for the cost calculation of the technologies involved in the Direct Air Carbon Capture and Storage ("DACCS") and Direct Air Carbon Capture and Utilization ("DACCU") pathways. To calculate the capital expenses (CAPEX) of water electrolysers in € kgH_2^{-1}, we assume an electrolyser lifetime of 20 years, a stack lifetime of 7 years, and 8760 hours of operation per year. Starred technologies indicate values on which a sensitivity analysis was performed.

Technology	CAPEX		Installed capacity	
	**2020**	**Ref.**	**2020**	**Ref.**
DAC*	870 € tCO_2^{-1}	[1]	10 $ktCO_2$	[2]
Polymer membrane eletrolysis	920 € kW^{-1}	[3]	7 MtH_2	[4]
Alkaline electrolysis	758 € kW^{-1}	[3,5]	7 MtH_2	[4]
Reverse-Water-Gas-Shift	4.98 € tCO^{-1}	[6,7]	0.002 MtCO	[4]
Electrochemical CO_2 reduction	421.53 € tCO^{-1}	[8,9]	0.002 MtCO	[4]
**Capital and operational costs assumed constant**				
Fischer-Tropsch synthesis CAPEX	0.108 € kg_{fuel}^{-1} assumed constant	[4]		

Supplementary Table 2 Mass flows for the relevant steps of the Direct Air Capture-based synthetic fuel production.

Mass	Process			
	**Electrochemical CO_2 reduction**	**Ref.**	**Reverse Water-Gas Shift**	**Ref.**
CO_2 per CO	1.572 $kgCO_2$ $kgCO^{-1}$	Calculated from sources below	1.57 $kgCO_2$ $kgCO^{-1}$	[8–10]

CO per fuel	1.967 $kgCO\ kg_{fuel}^{-1}$	[4]	1.969 $kgCO\ kg_{fuel}^{-1}$	[4,10]
Tot CO_2 per fuel	3.148 $kgCO_2\ kg_{fuel}^{-1}$	[4]	3.09 $kgCO_2\ kg_{fuel}^{-1}$	Calculated from sources above
H2 per CO		[11]	0.071 $kgH_2\ kgCO^{-1}$	[11,12]
H2 per FT			0.3 $kgH_2\ kg_{FT}^{-1}$	[4,10]
Tot H2 per fuel	0.297 $kH_2\ kg_{fuel}^{-1}$	[4]	0.438 $kgH_2\ kg_{fuel}^{-1}$	Calculated from sources above

Supplementary Table 3 Input parameters for the yearly energy consumption of the technologies involved in the Direct Air Carbon Capture and Storage (DACCS) and Direct Air Carbon Capture and Utilization (DACCU) pathways.

Technology	Electricity consumption				Heat consumption	
	2020	Ref.	2050	Ref.		Ref.
DAC	0.5 $MWh_e\ tCO_2^{-1}$	[13]	0.25	[4]	1.75 $MWh_{th}\ tCO_2^{-1}$	[14]
Polymer membrane eletrolysis	57 $kWh_e\ kg_{H2}^{-1}$	[3,15–19]	47.5 $kWh_e\ kg_{H2}^{-1}$			
Alkaline electrolysis	53 $kWh_e\ kg_{H2}^{-1}$	[3,4,22–25]	45.5 $kWh_e\ kg_{H2}^{-1}$			
Reverse-Water-Gas-Shift	0.11 $kWh_e\ kg_{CO}^{-1}$	[11]	0.11 $kWh_e\ kg_{CO}^{-1}$	[11]	0.49 $kWh_{th}\ kg_{CO}^{-1}$	[11]
Electrochemical CO_2 reduction	6.34 $kWh_e\ kg_{CO}^{-1}$	[4,8,9]	6.34 $kWh_e\ kg_{CO}^{-1}$	[4,8,9]		
Fischer-Tropsch synthesis	0.26 kWh kg_{fuel}^{-1}	[6]	0.26 kWh kg_{fuel}^{-1}	[6]		

Supplementary Table 4 Input parameters and basic assumptions to calculate increase in price per passenger per flight.

Parameter	Description		
Occupancy aircraft	80%		
Gross profit margin[26]	25%		
	**London - Berlin**	**London – New York**	**London - Perth**
Number passengers	178<br>With Boeing 787-9 Dreamliner	318.5<br>Average of Boeing 787-9 Dreamliner, Boeing 777-200LR, Boeing 777-3000LR, and Boeing 767-300	296 With Boeing 737-900
Distance flown (km)	964	5570	14500
Percentage of ticket price due to fuel[27]	25%	35%	45%

Supplementary Table 5 Input parameters and basic assumptions for the additional demand-side, contrails mitigation, and policy scenarios.

Parameter	Description
Decreasing demand	-2% yearly change in aviation demand (EJ)
Capped demand	+0% yearly change in aviation demand (EJ)
Rerouting	Reduction by 50% in contrails at a fuel increase by 1% based on the review by Dray et al.[28]
Ignoring contrails	Elimination of contrails formation assuming a mix of optimal rerouting and hydrogenation of fossil kerosene [29–31], without including the additional costs
Cost on CO_2 emissions	€100 tCO_2^{-1} in 2020 and then increase by 1% yearly
Cost on all aviation climate impacts	€100 tCO_{2e*}^{-1} in 2020 and then increase by 1% yearly
Subsidy on DACCS	€100 tCO_2^{-1} in 2020 and then decrease by 1% yearly
Subsidy on DACCU	€100 tCO_2^{-1} (€33 t_{fuel}^{-1}) in 2020 and then decrease by 1% yearly
Excess electricity	Electricty cost is 10% of basic assumption (€0.003 kWh^{-1})

Supplementary Figures

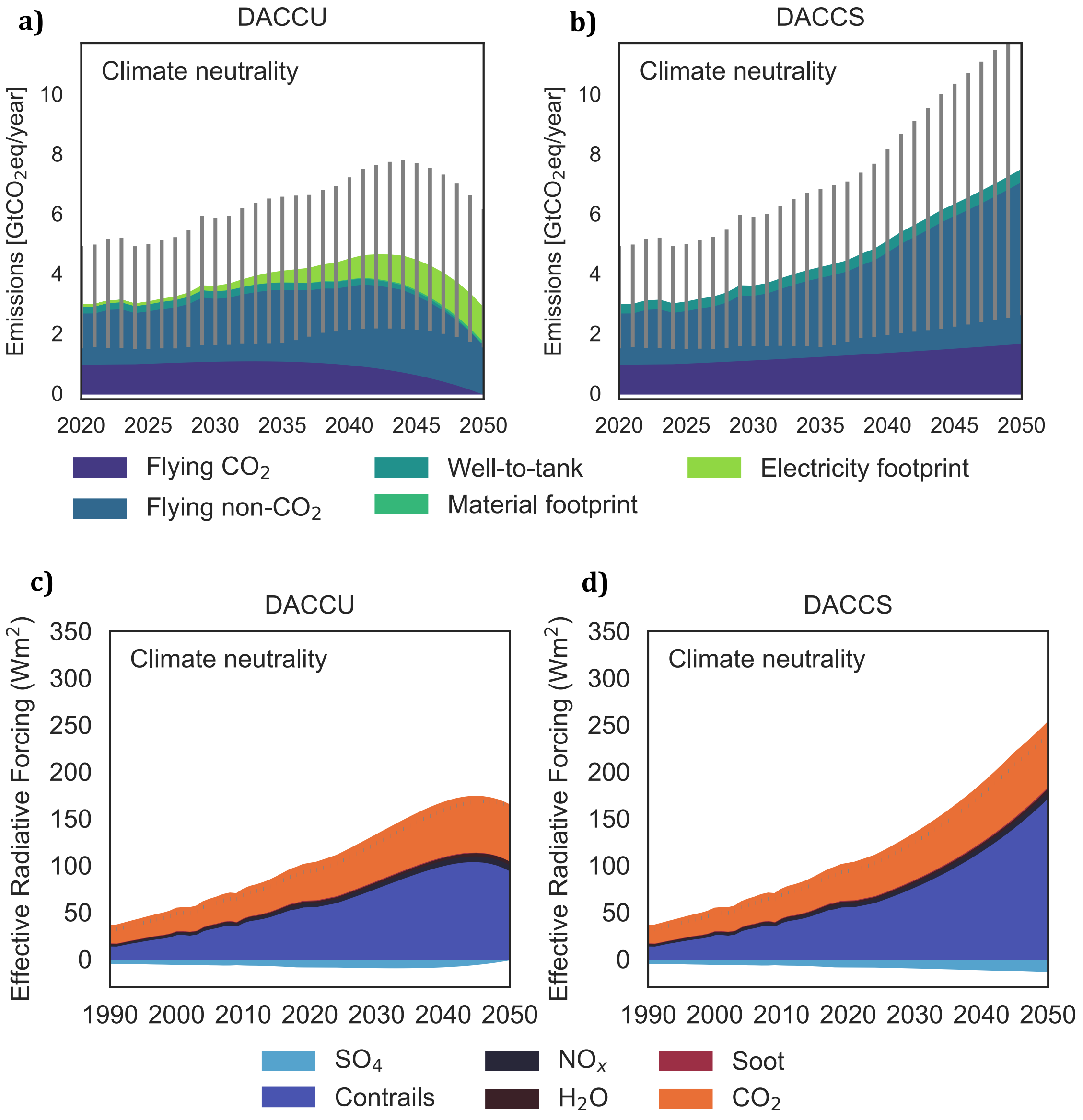

Supplementary Figure 1 a) Absolute emissions of Direct Air Carbon Capture and Utilization ("DACCU") and **b)** Direct Air Carbon Capture and Storage ("DACCS") pathways (excluding emission removals). Grey bars show uncertainties due to the non-CO_2 effects of aviation. **c)** Effective radiative forcing of the flight emissions of DACCU and **d)** DACCS pathways, broken down into their different components.

Supplementary Figure 1 shows the contrasting impacts of the Direct Air Carbon Capture and Utilization (DACCU) and Direct Air Carbon Capture and Storage (DACCS) pathways on direct aviation emissions and their effective radiative forcing (ERF). DACCU progressively eliminates CO_2 emissions, leading to a stabilization of their effective radiative forcing due to the long-lived nature of CO2 emissions. For most short-lived emissions and effects, such as condensation trails, DACCU actively reduces the effective radiative forcing, but does not completely eliminate these emissions. As demand for aviation continues to grow, the effective radiative forcing of non-CO2 species peaks around 2045, but then begins to decline. This reduction in emissions also applies

to historically 'cooling' emissions, such as sulphate aerosols (SO_4), which lose their negative effective radiative forcing by mid-century. Consequently, DACCU helps to stabilise the long-lived CO_2 forcing, while reducing - but not eliminating - the overall warming contribution from short-lived effects. In contrast, DACCS adopts an emit-and-remove strategy, resulting in a continuous increase in direct aviation emissions over time. This continued growth in emissions leads to a linear increase in effective radiative forcing, as DACCS focuses solely on removing CO_2 without addressing short-lived climate pollutants. As a result, DACCS is less effective in mitigating the wider climate impacts of aviation, particularly those caused by non-CO_2 emissions.

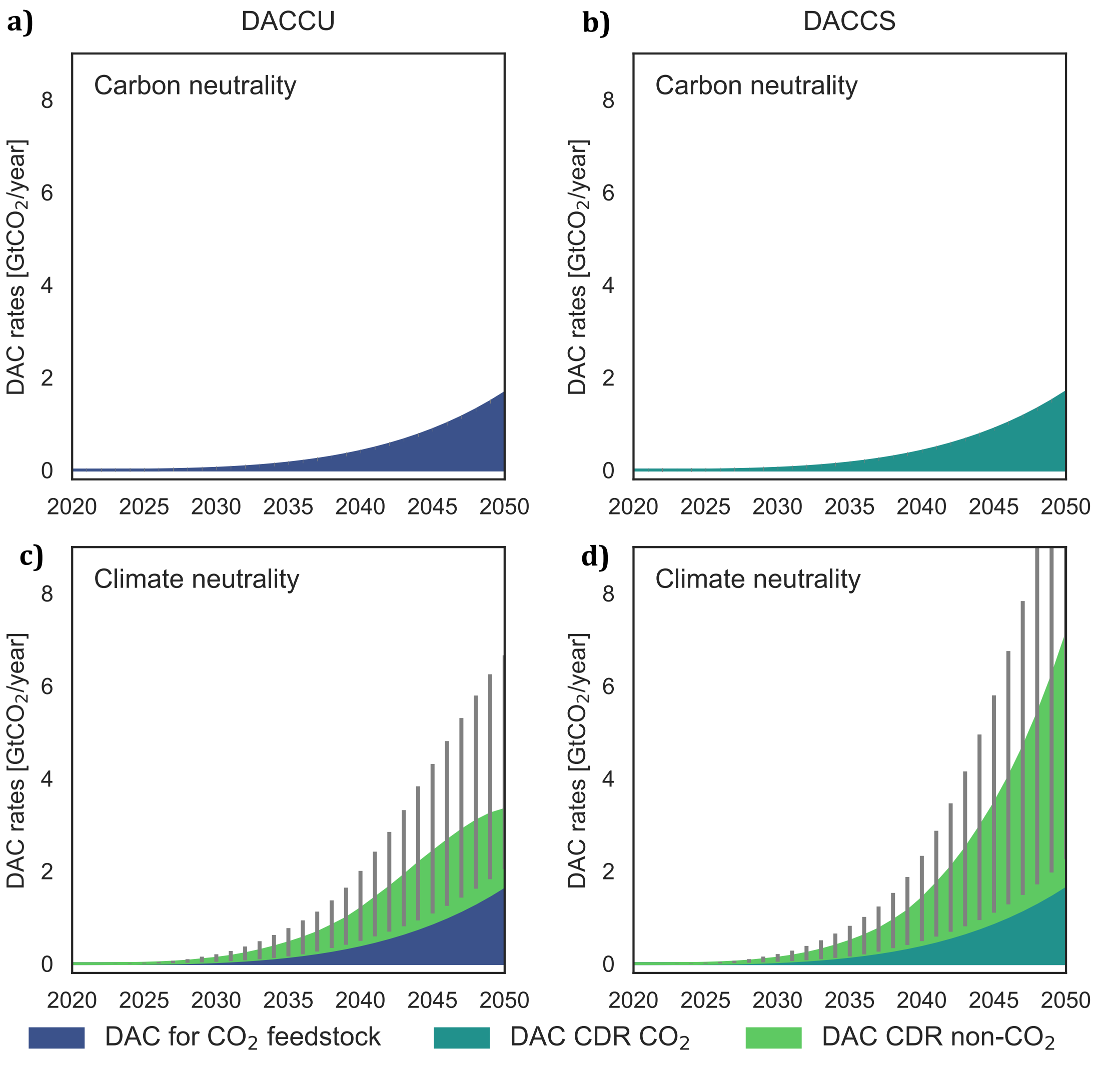

Supplementary Figure 2 Installed Direct Air Capture (DAC) rates under different climate targets and technology scenarios: **a)** Direct Air Carbon Capture and Utilization (DACCU) and CO_2 neutrality; **b)** Direct Air Carbon Capture and Storage (DACCS) and CO_2 neutrality; **c)** Direct Air Carbon Capture and Utilization (DACCU) and climate neutrality; **d)** Direct Air Carbon Capture and Storage (DACCS) and climate neutrality.

Supplementary Figure 2 illustrates the required Direct Air Capture (DAC) rates for both DACCU and DACCS under two scenarios: CO_2 neutrality and climate neutrality. For CO_2 neutrality, the DAC requirements are similar for both pathways. This is because both require the same amount of CO_2 emissions to be neutralized, regardless of whether the captured CO_2 is used to produce synthetic fuels (DACCU) or stored underground (DACCS). The focus is solely on offsetting CO_2, resulting in comparable DAC rates. Under climate neutrality, however, DAC needs to address

both CO_2 and non-CO_2 effects, such as condensation trails. DACCU reduces non-CO_2 emissions at source, so significantly less DAC is required to compensate for residual non-CO_2 emissions to achieve climate neutrality. In this pathway, DAC rates peak around 2045 and then stabilize, reflecting the decreasing need as non-CO_2 impacts are reduced. In contrast, DACCS does not address non-CO_2 emissions at source, requiring much higher DAC rates to compensate for their impact. The DAC requirements for DACCS grow exponentially, highlighting its limitations in achieving comprehensive climate neutrality.

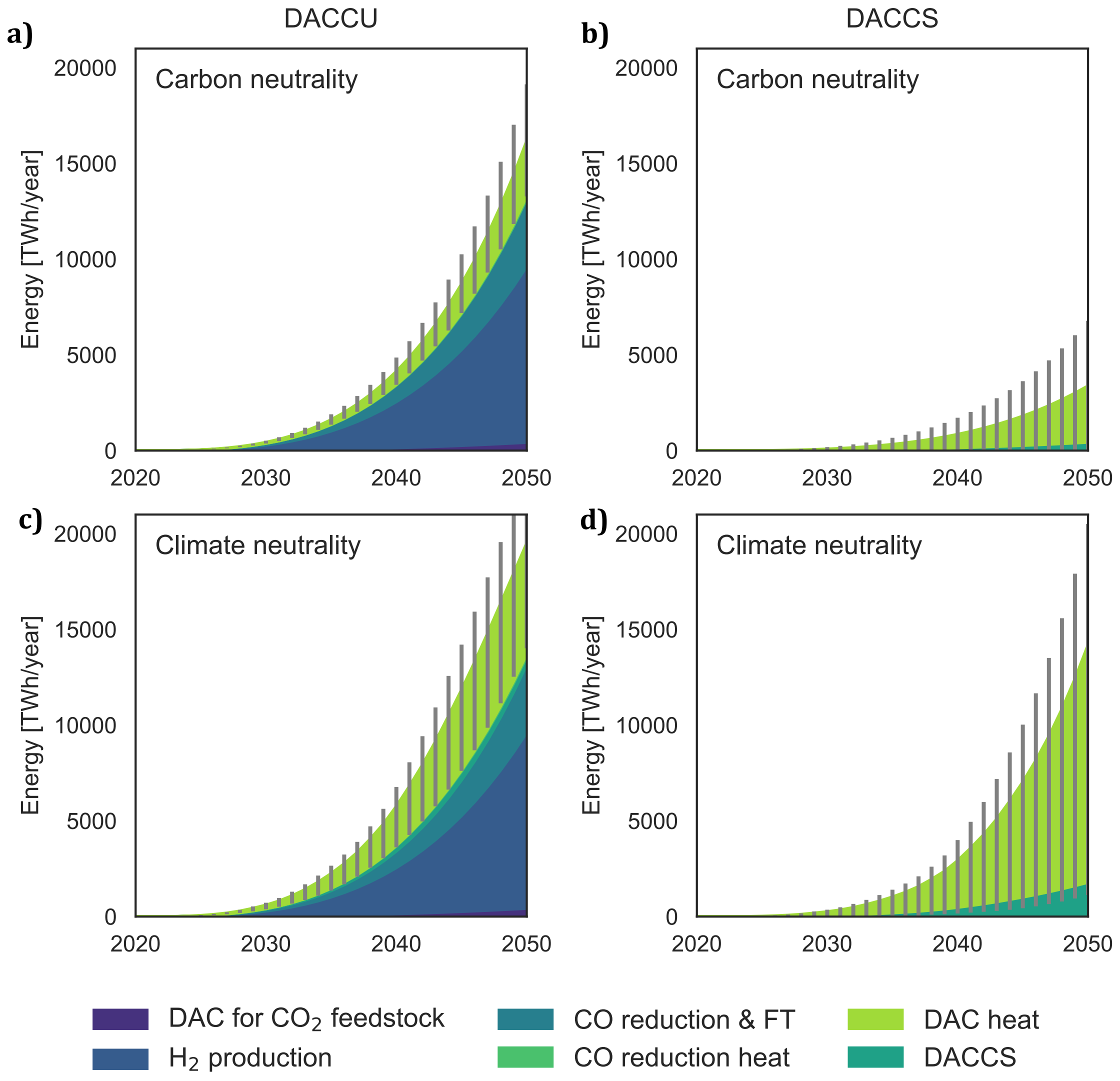

Supplementary Figure 3 Energy needed under different climate targets and technology scenarios: **a)** Direct Air Carbon Capture and Utilization (DACCU) and CO_2 neutrality; **b)** Direct Air Carbon Capture and Storage (DACCS) and CO_2 neutrality; **c)** Direct Air Carbon Capture and Utilization (DACCU) and climate neutrality; **d)** Direct Air Carbon Capture and Storage (DACCS) and climate neutrality.

Supplementary Figure 3 compares the energy requirements for DACCU and DACCS under CO_2 neutrality and climate neutrality. For CO_2 neutrality, DACCU requires significantly more energy due to the green hydrogen required for synthetic fuel production. In contrast, DACCS requires mainly heat for the capture process and uses relatively little energy to achieve CO_2 neutrality. For climate neutrality, the energy requirement for DACCS increases significantly as it has to compensate for non-CO_2 emissions. In contrast, DACCU maintains a high but stable energy requirement.

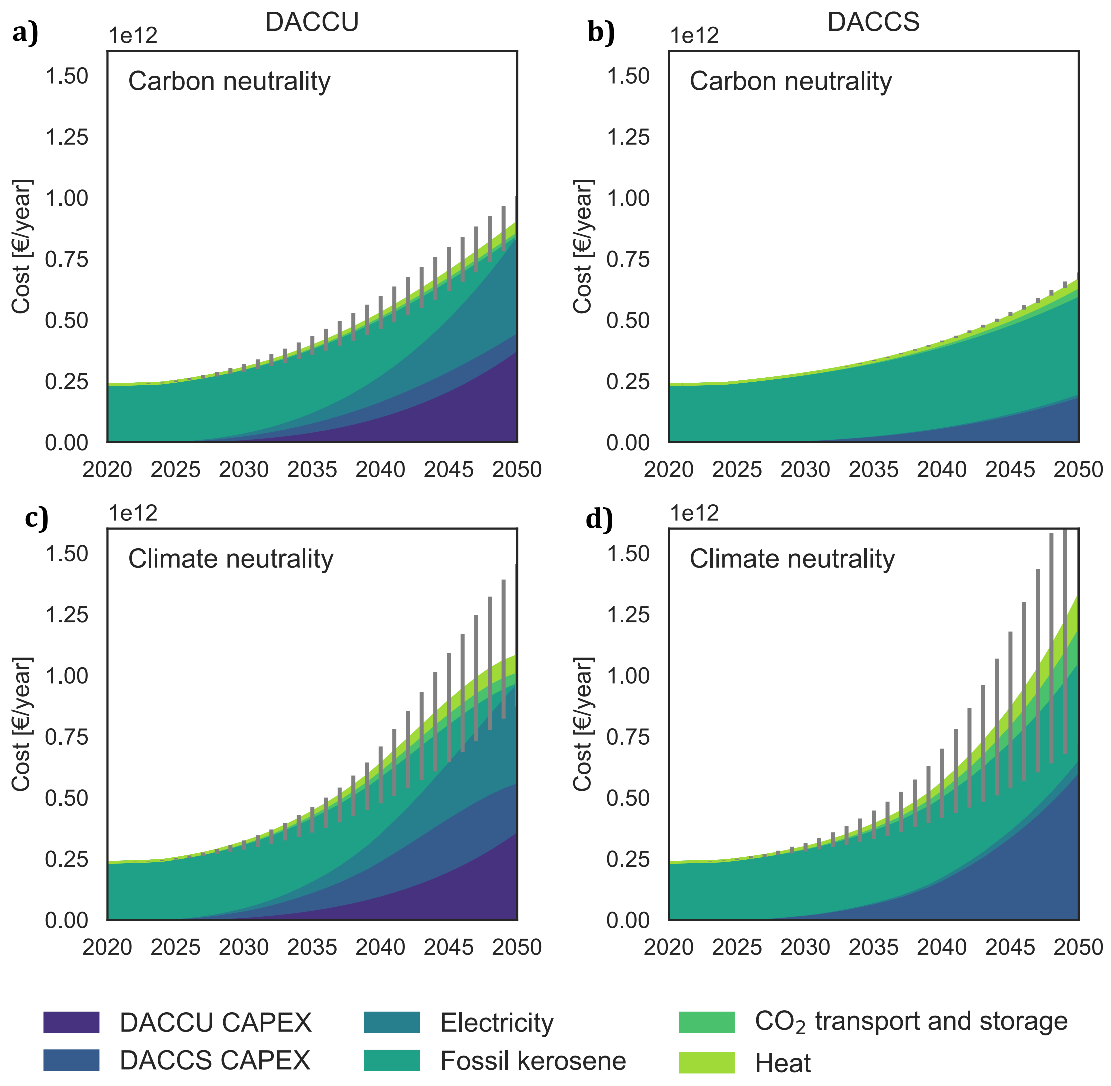

Supplementary Figure 4 Total cost of the two pathways until 2050 under different climate targets and technology scenarios: **a)** Direct Air Carbon Capture and Utilization (DACCU) and CO_2 neutrality; **b)** Direct Air Carbon Capture and Storage (DACCS) and CO_2 neutrality; **c)** Direct Air Carbon Capture and Utilization (DACCU) and climate neutrality; **d)** Direct Air Carbon Capture and Storage (DACCS) and climate neutrality.

Supplementary Figure 4 illustrates the evolution of cost components for DACCS and DACCU under CO_2 neutral and climate neutral scenarios. In both scenarios, fossil kerosene initially accounts for the largest share of costs, but the cost trajectories differ from 2030 onwards. Over time, in the DACCU scenario, these costs are progressively replaced by costs associated with DACCU, in particular capital expenditure (CAPEX) and electricity required for green hydrogen production. A smaller proportion of the cost comes from DACCS, which is used to offset lifecycle emissions under CO_2 neutrality and both lifecycle and non-CO_2 emissions under climate neutrality. This transition highlights DACCU's shift from fossil fuel dependency to renewable energy-based systems.

Fossil fuel costs remain significant throughout the DACCS scenarios. However, by 2050 under climate neutrality, around two-thirds of the total cost is driven by the capital expenditure

associated with DACCS to offset both CO_2 and non-CO_2 emissions. This reflects the increasing reliance on DACCS infrastructure to address the wider climate impacts of aviation.

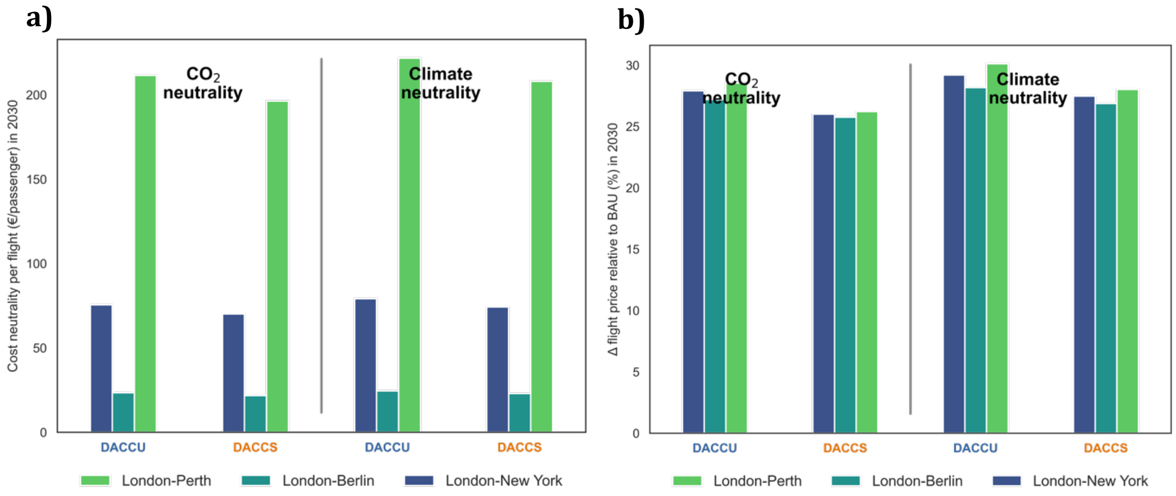

Supplementary Figure 5 Increase (**a**) and percentage change (**b**) in price per ticket due to additional Direct Air Capture-based mitigation in 2030.

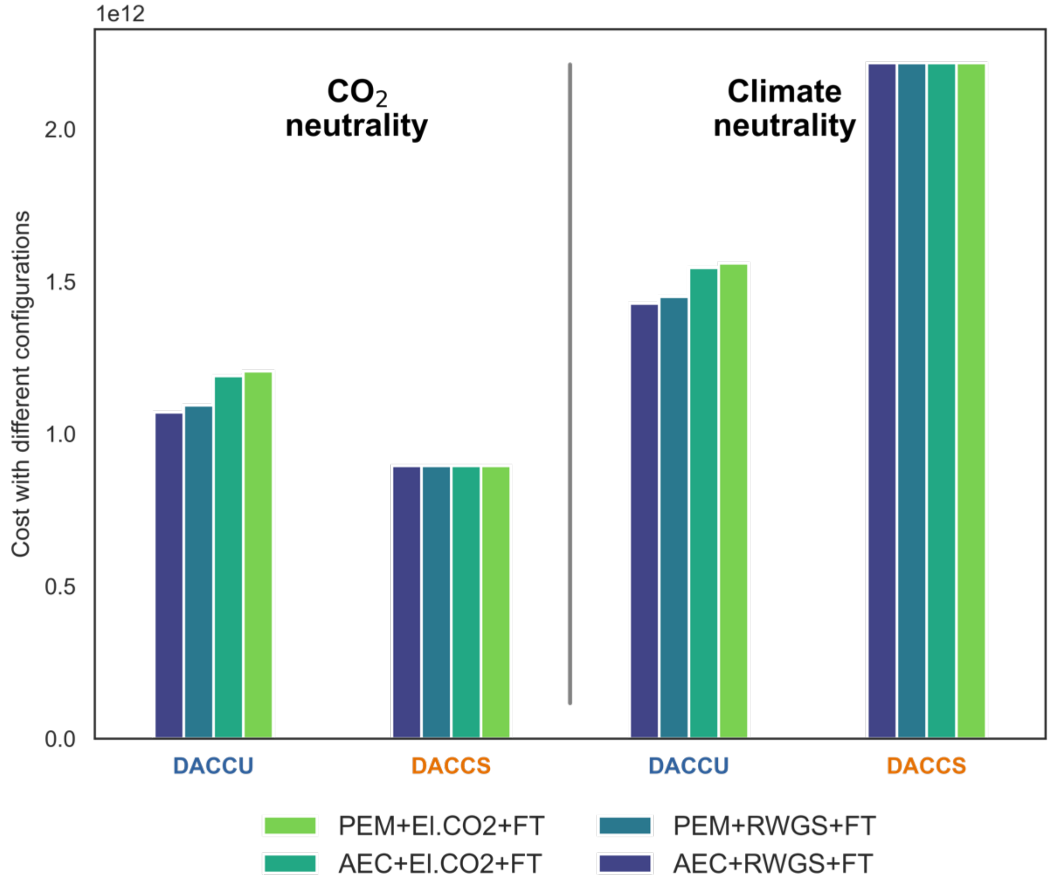

Supplementary Figure 6 Final cost by 2050 in € for different configurations of Direct Air Carbon Capture and

Utilization, resulting from the different combination of polymer membrane electrolysis (PEM), alkaline electrolysis (AEC), electrochemical CO_2 reduction (El.CO_2), reverse-water-gas-shift (RWGS), and Fischer-Tropsch synthesis (FT).

Supplementary Figure 5 shows the total cost to 2050 of achieving CO_2 and climate neutrality under different technology configurations. The use of Reverse Water Gas Shift (RWGS) significantly reduces costs compared to electrochemical CO This is due to the lower energy requirements of RWGS and its cost-effective integration with renewable energy sources. In addition, the use of alkaline electrolysis instead of polymer membrane electrolysis offers further cost savings, although the impact is comparatively smaller. In total, the cost savings from the technology choices amount to up to 1 million, or about 10% of the total cost.

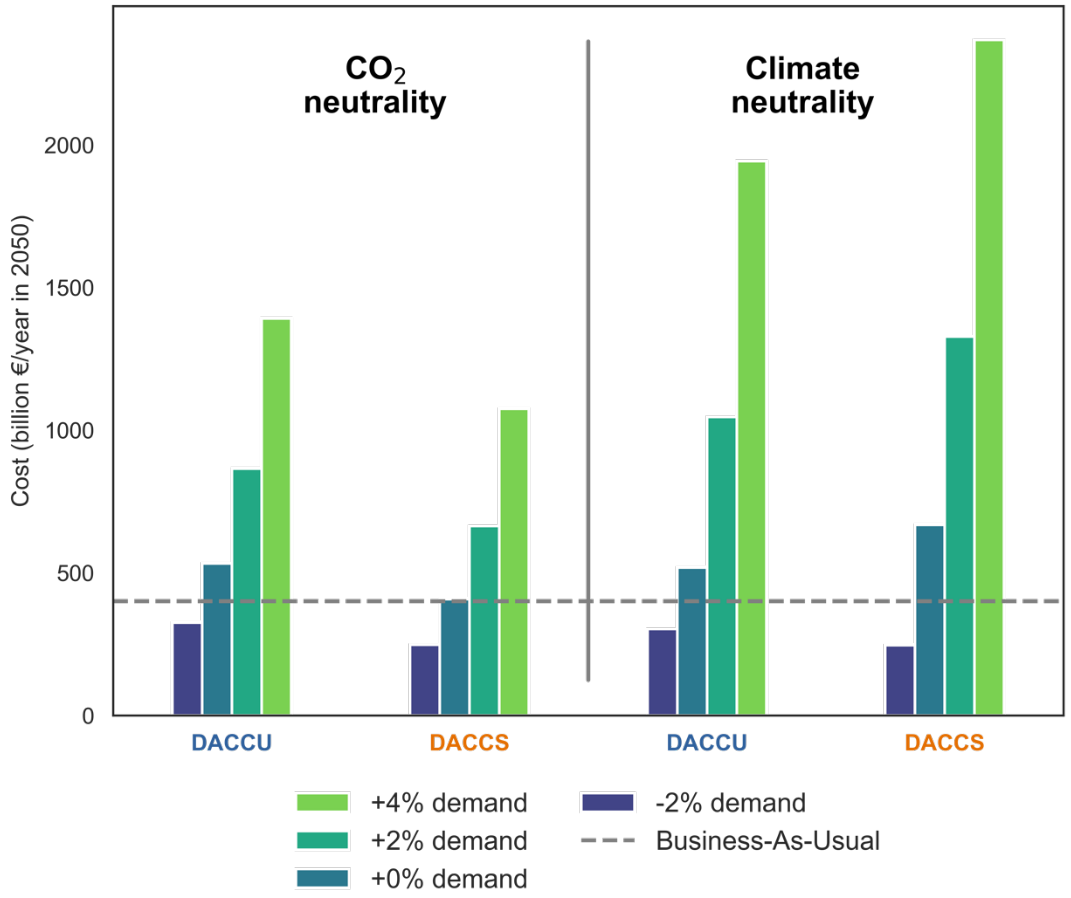

Supplementary Figure 7 Final cost by 2050 in € for different demand scenarios.

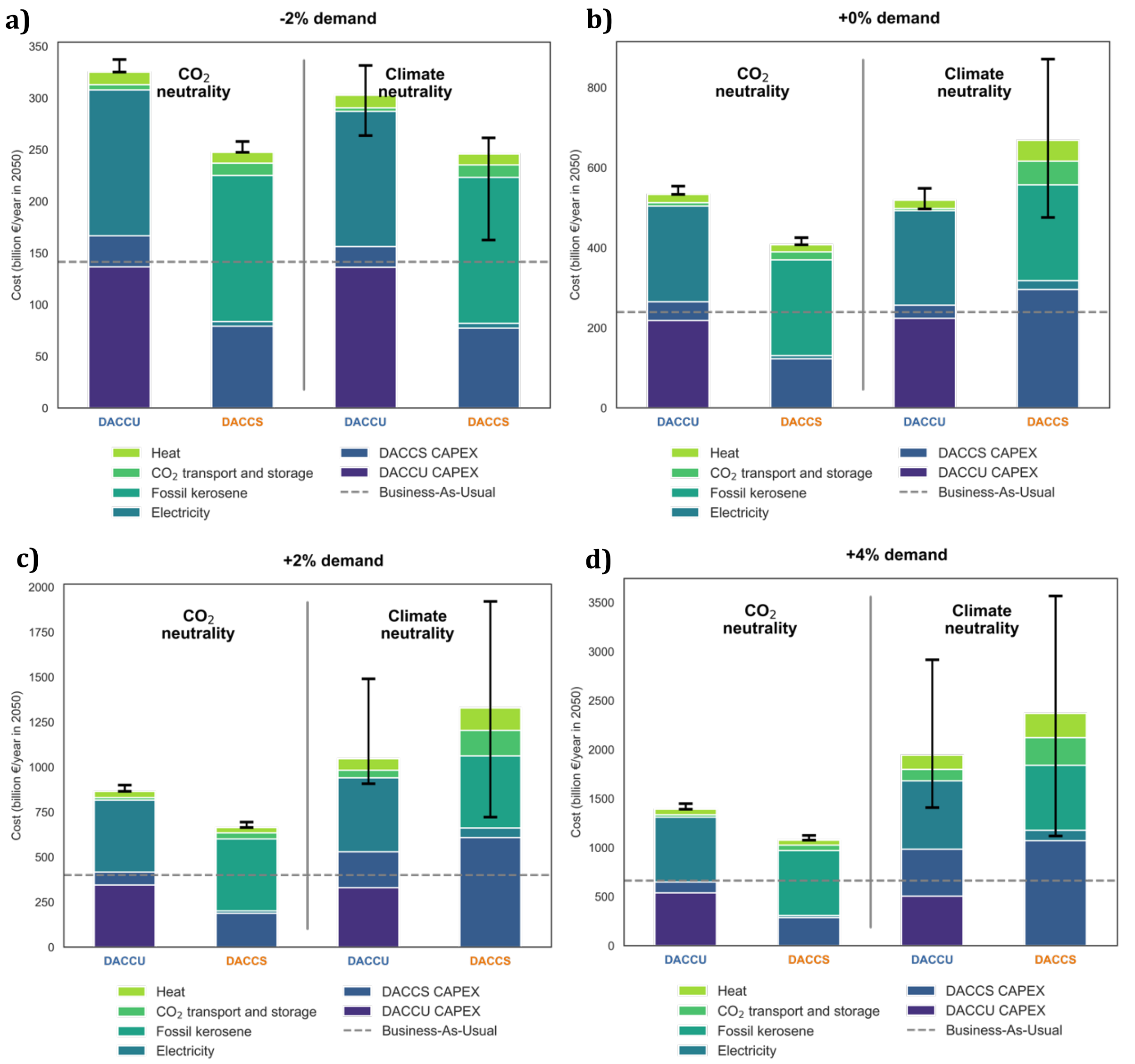

Supplementary Figure 8 Breakdown of final cost by 2050 in € for different demand scenarios: **a)** decreasing demand (-2% demand change yearly); **b)** stagnating demand (0% demand change yearly); **c)** target growth in demand from International Civil Aviation Organization (+2% demand change yearly); **d)** historical growth in demand (+4% demand change yearly).

Supplementary Figures 7 and 8 explore the total and disaggregated costs of achieving CO_2 and climate neutrality in 2050 using DACCU and DACCS under different global aviation demand scenarios. Lower demand scenarios result in significantly lower total costs compared to a business-as-usual trajectory. This highlights the cost-saving potential of demand reduction as a complementary strategy to technological solutions. The demand scenario also affects the relative cost-effectiveness of DACCU and DACCS. At higher demand levels, DACCU becomes increasingly advantageous for achieving climate neutrality due to its ability to address both CO_2 and non-CO_2 emissions. In contrast, DACCS faces increasing costs under higher demand scenarios, driven by the growing need for CO_2 removal to offset aviation emissions. Interestingly, with DACCU achieving climate neutrality is less expensive than achieving CO_2 neutrality in low demand cases. This is because the short-lived climate pollutants addressed by DACCU - such as condensation trails and aerosols - have a pronounced cooling effect under reduced demand. These cooling effects reduce the need for further CO_2 offsets, lowering the overall cost of achieving climate neutrality compared to CO_2 neutrality.

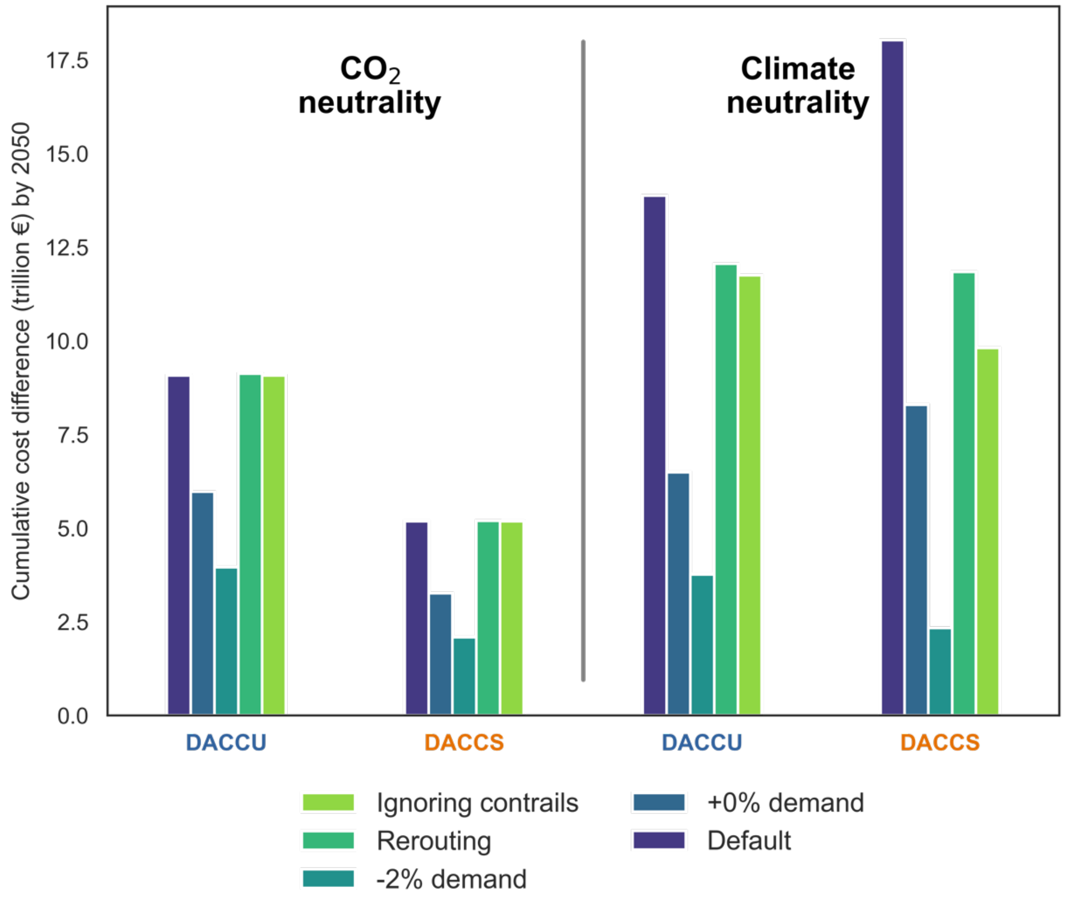

Supplementary Figure 9 Cumulative difference in cost relative to a business-as-usual under different demand and contrail mitigation scenarios for the Direct Air Carbon Capture and Utilization (DACCU) and the Direct Air Carbon Capture and Storage (DACCS) under a CO_2 and climate neutrality target.

Supplementary Figure 9 shows the cumulative cost difference compared to a business-as-usual scenario under different demand and contrail mitigation strategies. For CO_2 neutrality, DACCS consistently results in lower cumulative costs than DACCU due to its simpler implementation and focus on long-lived emissions. For climate neutrality, the cost-effectiveness of DACCU versus DACCS varies depending on demand and contrail mitigation measures. Under high demand or scenarios involving rerouting to mitigate contrails, DACCU proves superior due to its ability to effectively address both CO_2 and non-CO_2 emissions. However, with more significant demand reductions or aggressive contrail mitigation strategies, the cost advantage of DACCU diminishes and DACCS becomes equally competitive.

a)

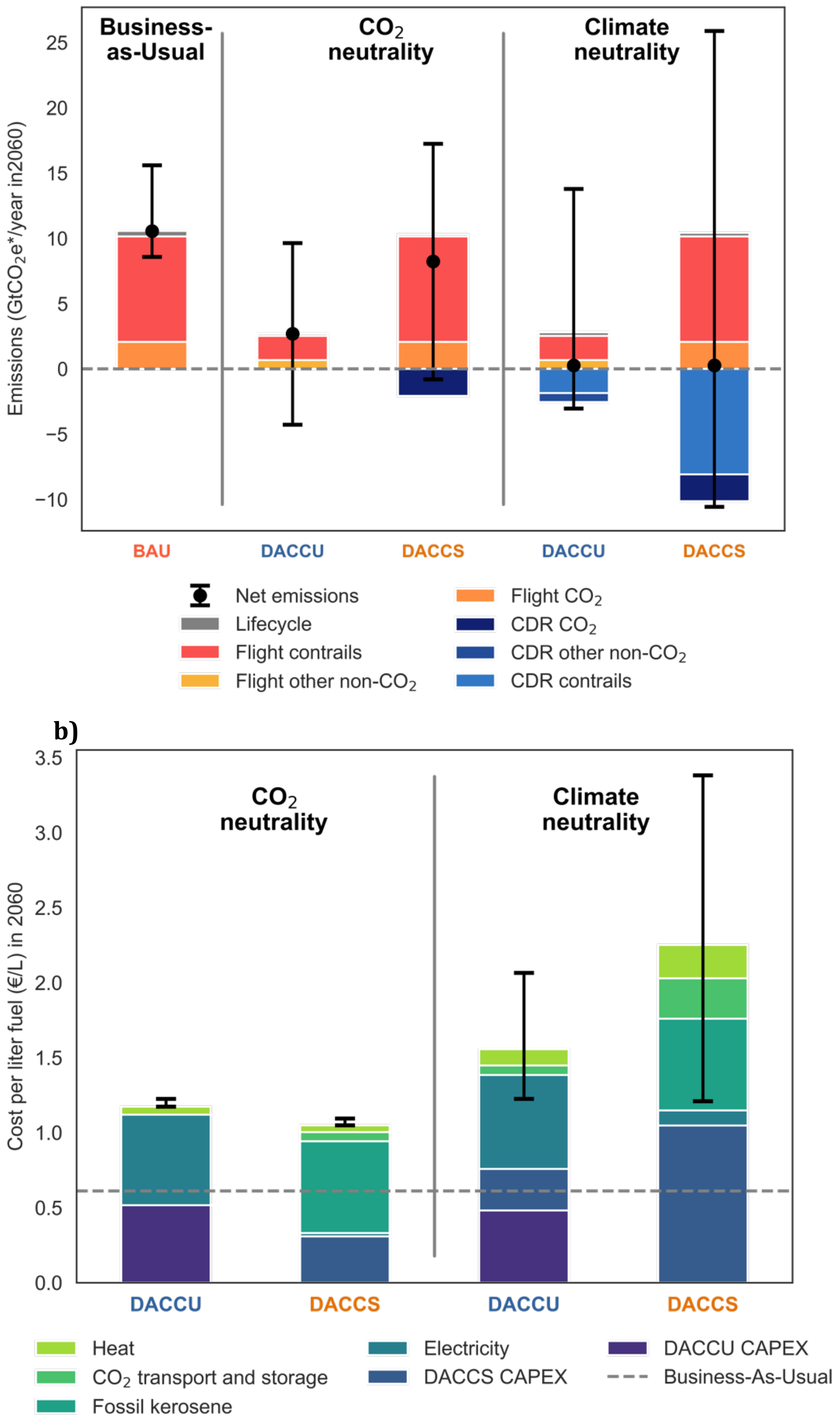

Supplementary Figure 10 a) Emissions and **b)** cost per liter fuel to achieve CO_2 and climate neutrality in 2060.

Supplementary Figure 10 shows the emissions (a) and cost per liter of fuel (b) required to achieve CO_2 and climate neutrality by 2060. In 2060, the cost difference between DACCS and DACCU for CO_2 neutrality is smaller than in 2050. This is due to continuous cost reductions in

the technologies associated with DACCU, although its emission reductions remain the same as in 2050. In contrast, the DACCS pathway faces increasing CO_2 emissions, driving up the cost of achieving net-zero CO_2. The same mechanisms apply to climate neutrality. DACCU maintains the same level of emissions as in 2050, while emissions in the DACCS pathway continue to increase. This results in significantly lower costs for DACCU compared to the DACCS pathways due to its less effective treatment of short-lived climate pollutants. These trends highlight the long-term cost advantage of DACCU under climate neutrality, while under CO_2 neutrality the narrowing cost gap underlines the increasing challenges faced by DACCS due to rising emissions.

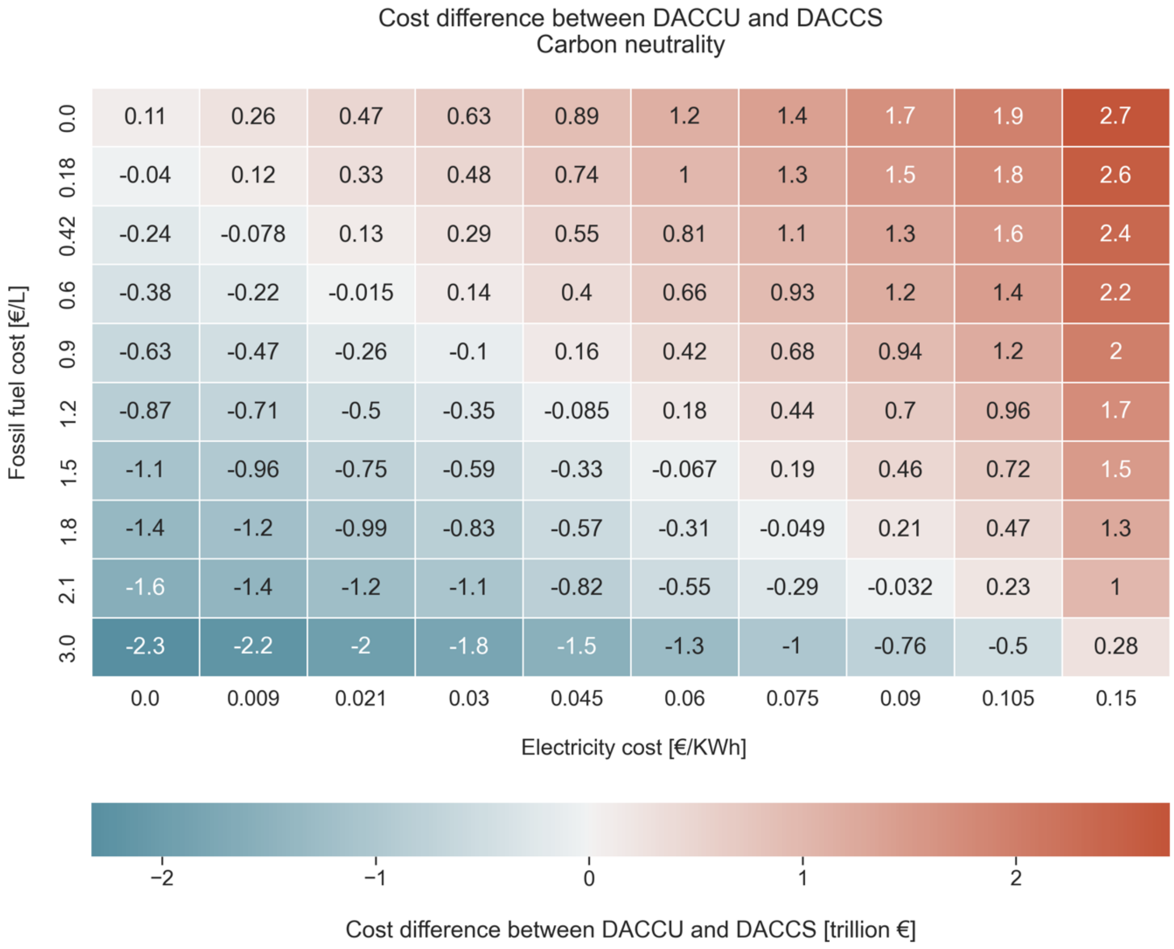

Supplementary Figure 11 Impact of electricity cost and fossil kerosene cost on the difference in cost by 2050 of DACCS and DACCU pathways to reach CO_2 neutrality. Each cell shows the difference in cost between DACCU and DACCS pathways under varying assumptions on the learning rate (y-axis) and fossil kerosene cost (x-axis).

Supplementary Figure 11 examines the impact of electricity and fossil fuel costs on the cost differential between DACCS and DACCU pathways to achieve CO_2 neutrality by 2060. A combination of low electricity prices and high fossil fuel costs could make DACCU competitive with DACCS, even at CO_2 neutrality. If subsidies for fossil fuel production were removed, fossil kerosene could cost around €0.9 per liter. In such a scenario, cost parity between DACCU and DACCS could be achieved if electricity costs were around €0.03 per kWh - a price level consistent with recent observations for wind power.

a)

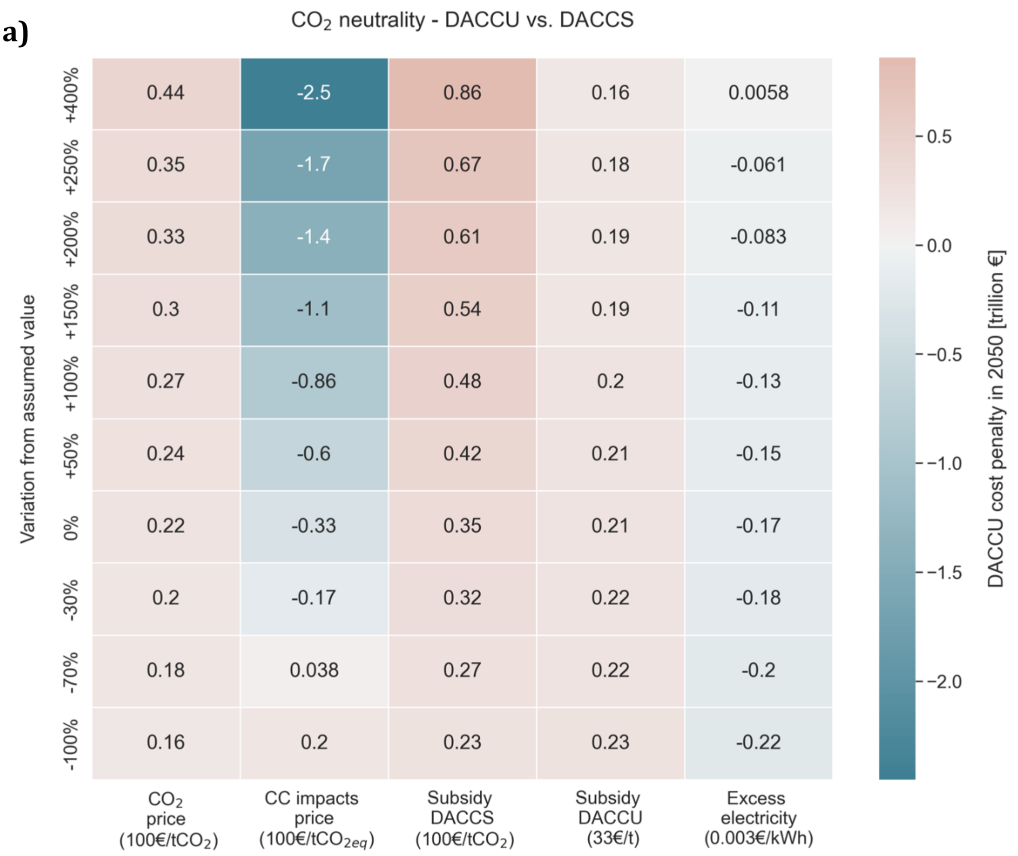

b)

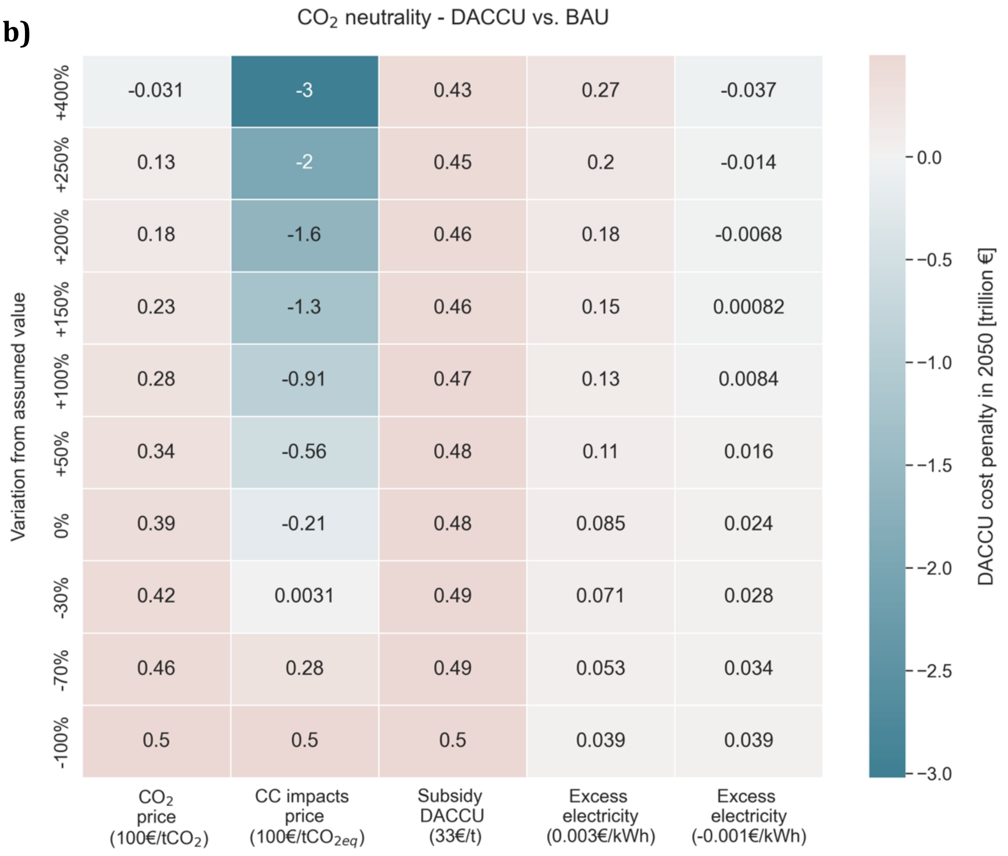

Supplementary Figure 12 Impact of varying assumptions on different policies on the difference in cost by 2050 of **a)** Direct Air Carbon Capture and Storage (DACCS) and Direct Air Carbon Capture and Utilization (DACCU) pathways to reach CO_2 neutrality and **b)** DACCU and fossil jet fuels assuming a 100%

dominance of each fuel type by 2050. The row with 0% represents the standard assumption about how the policy is implemented, namely a price on CO_2 emissions by 100€ tCO_2^{-1}, a price on aviation climate impacts by 100€ tCO_{2eq}^{-1}, a subsidy to DACCS by 100€ tCO_2^{-1}, a subsidy to DACCU by 33 € t^{-1} synthetic fuel, or a restricted use of excess electricity of a price by 0.003€ kWh^{-1}. The other rows represent variation of this input assumptions on the policy value (e.g., by -70% the price on CO_2 emissions will be 30€ tCO_2^{-1}, while by +400% it will be 500€ tCO_2^{-1}).

Supplementary Figure 12 shows the cost differences between DACCU and DACCS (a) and between DACCU and fossil jet fuel (b) under different policy scenarios. Pricing of emissions is key to reducing the cost gap. Internalising the environmental costs of fossil jet fuel and lifecycle emissions for DACCU and DACCS aligns fossil fuel prices with their climate impacts. However, pricing CO_2 emissions alone cannot make DACCU cost-competitive with DACCS under CO_2 neutrality, as it primarily applies to indirect emissions, which are higher for DACCU. Conversely, pricing all aviation-related climate impacts significantly favours DACCU, making it cheaper than DACCS at only €30 per tonne of CO_2 equivalent (tCO_2^{e*}). To compete with fossil jet fuels, emission prices would have to exceed €500 per tCO_2 or €100 per tCO_2e^* for all climate impacts. Subsidies for the production of synthetic fuels, even at €500 per tonne, are insufficient to close the cost gap. A more effective strategy is to exploit electricity costs below €0.01 kWh^{-1} during surplus periods through seasonal restrictions on the production of DACCU-based fuels. While this may limit production volumes and increase capital expenditure per unit, it could also reduce wear and tear on expensive components, thereby extending their life.

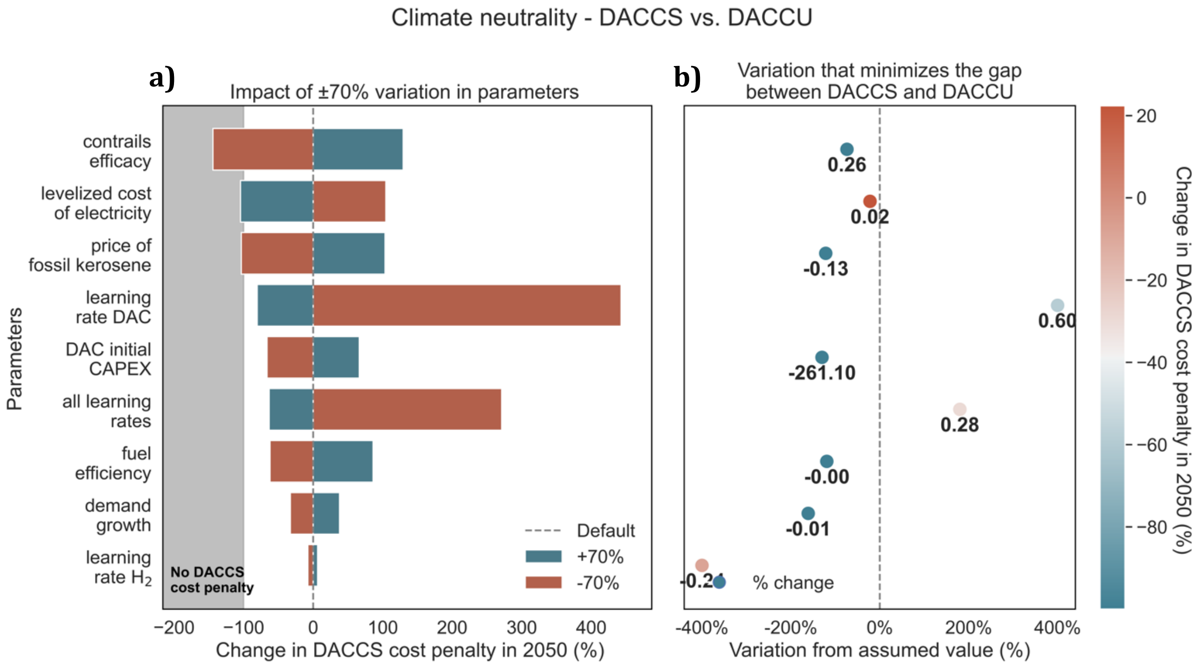

Supplementary Figure 13 Impact of local variation in key input parameters on the DACCS cost penalty relative to DACCU to achieve climate neutrality by 2050. Panels **(a)** describes the impact of ±100% variation in input parameters on the change in DACCU cost penalty by 2050 (%). The shaded gray area indicates where the DACCU cost penalty is eliminated i.e., DACCU becomes cost advantageous compared to either DACCS or business as usual. Panels **(b)** shows the "optimized" values of the input parameters, i.e., the values at which the cost penalty is minimized (where the difference in total cost between DACCU and either DACCS or business as usual is closest to zero). The points on the x-axis show the percentage

change from the default value, with the numbers in the figure showing the new "optimal" values. The colors of the points represent the extent to which the optimized value closes the price gap, corresponding to a change in the DACCU cost penalty by 2050.

Supplementary Figure 13 shows the impact of local variations in key input parameters on the cost penalty of DACCU compared to DACCS in achieving climate neutrality by 2050. Varying the parameters by ±70% only affects the cost penalty for contrail efficiency, electricity price and fossil fuel kerosene price. In particular, reducing contrail effectiveness and kerosene price or increasing electricity price reduces the cost gap. Contrail effectiveness is the most influential factor; it is the only parameter that can not only close the cost gap, but also make DACCS cheaper than DACCU. Cost parity between DACCS and DACCU can be achieved under alternative values of all factors except levelized cost of electricity and learning rates. For example, a contrail efficiency of 26% of the current best estimate, no fuel efficiency improvements for future aircraft, or a 1% annual reduction in demand could make DACCS as cost effective as DACCU. This highlights the importance of contrail mitigation and demand management in shaping the relative cost competitiveness of these pathways.

Supplementary reference

1. Young, J. *et al.* The cost of direct air capture and storage can be reduced via strategic deployment but is unlikely to fall below stated cost targets. *One Earth* **0**, (2023).
2. Smith, S. M. *et al. The State of Carbon Dioxide Removal - 1st Edition*. http://dx.doi.org/10.17605/OSF.IO/W3B4Z (2023) doi:10.17605/OSF.IO/W3B4Z.
3. IRENA. *Making the Breakthrough: Green Hydrogen Policies and Technology Costs*. https://www.irena.org/-/media/Files/IRENA/Agency/Publication/2020/Nov/IRENA_Green_Hydrogen_breakthrough_2021.pdf?la=en&hash=40FA5B8AD7AB1666EECBDE30EF458C45EE5A0AA6 (2021).
4. Becattini, V., Gabrielli, P. & Mazzotti, M. Role of Carbon Capture, Storage, and Utilization to Enable a Net-Zero-CO2-Emissions Aviation Sector. *Ind. Eng. Chem. Res.* **60**, 6848–6862 (2021).
5. Christensen, A. *Assessment of Hydrogen Production Costs from Electrolysis: United States and Europe*. https://theicct.org/publication/assessment-of-hydrogen-production-costs-from-electrolysis-united-states-and-europe/ (2020).
6. Terwel, R. & Kerkhoven, J. *Carbon Neutral Aviation with Current Enginge Technology: The Take-off of Synthetic Kerosene Production in the Netherlands*. 62 . https://kalavasta.com/assets/ reports/Kalavasta_Carbon_Neutral_Aviation.pdf (2018).

7. Zang, G. *et al.* Synthetic Methanol/Fischer–Tropsch Fuel Production Capacity, Cost, and Carbon Intensity Utilizing CO2 from Industrial and Power Plants in the United States. *Environ. Sci. Technol.* **55**, 7595–7604 (2021).
8. Jouny, M., Luc, W. & Jiao, F. General Techno-Economic Analysis of CO2 Electrolysis Systems. *Ind. Eng. Chem. Res.* **57**, 2165–2177 (2018).
9. Shin, H., Hansen, K. U. & Jiao, F. Techno-economic assessment of low-temperature carbon dioxide electrolysis. *Nat. Sustain.* **4**, 911–919 (2021).
10. Moretti, C. Reflecting on the environmental impact of the captured carbon feedstock. *Sci. Total Environ.* **854**, 158694 (2023).
11. Doty, F. D., Doty, G. N., Staab, J. P. & Holte, L. L. Toward Efficient Reduction of CO2 to CO for Renewable Fuels. in 775–784 (American Society of Mechanical Engineers Digital Collection, 2010). doi:10.1115/ES2010-90362.
12. Van der Giesen, C., Kleijn, R. & Kramer, G. J. Energy and Climate Impacts of Producing Synthetic Hydrocarbon Fuels from CO2. *Environ. Sci. Technol.* **48**, (2014).
13. Terlouw, T., Treyer, K., Bauer, C. & Mazzotti, M. Life Cycle Assessment of Direct Air Carbon Capture and Storage with Low-Carbon Energy Sources. *Environ. Sci. Technol.* **55**, 11397–11411 (2021).
14. Gabrielli, P., Gazzani, M. & Mazzotti, M. The Role of Carbon Capture and Utilization, Carbon Capture and Storage, and Biomass to Enable a Net-Zero-CO2 Emissions Chemical Industry. *Ind. Eng. Chem. Res.* **59**, 7033–7045 (2020).
15. Schmidt, P., Weindorf, W., Roth, A., Batteiger, V. & Riegel, F. *Power-to-Liquids: Potentials and Perspectives*. (2016).
16. Sutter, D., van der Spek, M. & Mazzotti, M. 110th Anniversary: Evaluation of CO2-Based and CO2-Free Synthetic Fuel Systems Using a Net-Zero-CO2-Emission Framework. *Ind. Eng. Chem. Res.* **58**, 19958–19972 (2019).
17. Yates, J. *et al.* Techno-economic Analysis of Hydrogen Electrolysis from Off-Grid Stand-Alone Photovoltaics Incorporating Uncertainty Analysis. *Cell Rep. Phys. Sci.* **1**, 100209 (2020).

18. Kopp, M. *et al.* Energiepark Mainz: Technical and economic analysis of the worldwide largest Power-to-Gas plant with PEM electrolysis. *Int. J. Hydrog. Energy* **42**, 13311–13320 (2017).

19. Alfian, M. & Purwanto, W. W. Multi-objective optimization of green urea production. *Energy Sci. Eng.* **7**, 292–304 (2019).

20. Schmidt, P., Batteiger, V., Roth, A., Weindorf, W. & Raksha, T. Power-to-Liquids as Renewable Fuel Option for Aviation: A Review. *Chem. Ing. Tech.* **90**, 127–140 (2018).

21. Nosherwani, S. A. & Neto, R. C. Techno-economic assessment of commercial ammonia synthesis methods in coastal areas of Germany. *J. Energy Storage* **34**, 102201 (2021).

22. Schmidt, O. *et al.* Future cost and performance of water electrolysis: An expert elicitation study. *Int. J. Hydrog. Energy* **42**, 30470–30492 (2017).

23. Treyer, K., Sacchi, R. & Bauer, C. *Life Cycle Assessment of Synthetic Hydrocarbons for Use as Jet Fuel: 'Power-to-Liquid' and 'Sun-to-Liquid' Processes.* (2022).

24. Liu, C. M., Sandhu, N. K., McCoy, S. T. & Bergerson, J. A. A life cycle assessment of greenhouse gas emissions from direct air capture and Fischer–Tropsch fuel production. *Sustain. Energy Fuels* **4**, 3129–3142 (2020).

25. Rosental, M., Fröhlich, T. & Liebich, A. Life Cycle Assessment of Carbon Capture and Utilization for the Production of Large Volume Organic Chemicals. *Front. Clim.* **2**, (2020).

26. Damodaran, A. Operating and Net Margins - Stern School of Business, New York University. https://pages.stern.nyu.edu/~adamodar/New_Home_Page/datafile/margin.html (2024).

27. Ringbeck, J., Gautam, A. & Pietsch, T. Endangered Growth: How the Price of Oil Challenges International Travel & Tourism Growth. in *The Travel & Tourismus Competitiveness Report 2009* 525 (World Economic Forum, 2009).

28. Dray, L. *et al.* Cost and emissions pathways towards net-zero climate impacts in aviation. *Nat. Clim. Change* **12**, 956–962 (2022).

29. Teoh, R., Schumann, U., Majumdar, A. & Stettler, M. E. J. Mitigating the Climate Forcing of Aircraft Contrails by Small-Scale Diversions and Technology Adoption. *Environ. Sci. Technol.* (2020) doi:10.1021/acs.est.9b05608.

30. Teoh, R. *et al.* Targeted Use of Sustainable Aviation Fuel to Maximize Climate Benefits. *Environ. Sci. Technol.* **56**, 17246–17255 (2022).

31. Transport & Environment. The easy fix to air pollution linked to planes. *Transport & Environment* https://www.transportenvironment.org/articles/the-easy-fix-to-air-pollution-linked-to-planes (2024).
